# Surgical Synechiolysis of Iridocapsular Adhesion and Sulcus Placement of a Rigid Intraocular Lens on an Oversized Residual Capsular Rim

**DOI:** 10.1155/2018/3068756

**Published:** 2018-09-12

**Authors:** Jiao Lyu, Qi Zhang, Haiying Jin, Tingyi Liang, Jili Chen, Peiquan Zhao

**Affiliations:** ^1^Department of Ophthalmology, Xinhua Hospital, School of Medicine, Shanghai Jiao Tong University, Shanghai, China; ^2^Shibei Hospital, Jing'an District, Shanghai, China

## Abstract

**Purpose:**

To report the surgical outcomes of surgical synechiolysis of iridocapsular adhesion and sulcus placement of a polymethyl methacrylate scleral-sutured intraocular lens (IOL) in aphakic eyes with an oversized residual capsular rim.

**Methods:**

Eight aphakic eyes from eight consecutive patients were studied retrospectively. Synechiolysis was performed to maximally expose the residual capsulorhexis. Then, the rigid IOL was placed on the preserved capsulorhexis into the ciliary sulcus.

**Results:**

Synechiolysis of iridocapsular adhesion was achieved in all eight eyes intraoperatively. Six eyes had extensive dissection to facilitate IOL sulcus placement. Consequently, seven of the eight eyes had the IOL secured by the residual capsulorhexis, and the other eye had the IOL haptics supported by the narrow residual capsular rim. A visual acuity of 0.25 or above was achieved in four of eight patients, and a well-centered IOL was observed in seven of the eight eyes 26 to 53 months after surgery. A mild IOL decentration was detected in the eye whose capsular rim was not securing the IOL optic.

**Conclusions:**

A large-optic and rigid IOL in the sulcus is a feasible alternative when a sulcus-based IOL is considered for aphakic eyes with an oversized residual capsulorhexis. A preserved capsulorhexis after sufficient synechiolysis, which can secure the IOL optic intraoperatively, may yield better stability of the IOL position.

## 1. Introduction

Sulcus placement of a posterior chamber intraocular lens (PCIOL) is a surgical alternative to correct aphakia in the presence of a deficient or absent posterior capsule [1, 2]. The key characteristics for a sulcus-based PCIOL include (1) a large optic and tip-to-tip size for lateral stability; (2) long thin angulated haptics for iris clearance; and (3) an IOL made from a foldable material that allows for a smaller incision [1, 3]. However, a foldable IOL may not be appropriate for sulcus placement in aphakic eyes with an oversized residual capsular rim [1]. These aphakic eyes usually have experienced a complicated cataract surgery, an ocular trauma, or multiple intraocular surgical interventions, leaving an irregular or noncontinuous capsulorrhexis or vitrectorrhexis, iridolenticular synechia, fibrosis, traction of ciliary process, and/or vitreoretinopathies. Thus, implantation of a sulcus-based PCIOL may involve synechiolysis, dissection of ciliary sulcus, insertion of a proper IOL on the preserved capsule tissue, and management of posterior segment comorbidities. Sulcus placement of a small size, relatively soft, foldable IOL may result in IOL decentration or dislocation, pigment dispersion syndrome, pigmentary glaucoma, or uveitis-glaucoma-hyphema syndrome [1, 4].

Surgical preservation of residual capsulorhexis and reconstruction of the ciliary sulcus are necessary for the stability of a sulcus IOL. Dissection techniques utilize a sharp dissection with a needle tip and capsulotomy vannas scissors or blunt dissection with viscoelastic material. However, it is difficult to achieve a 360° exposure of capsulorhexis almost to the ciliary sulcus, not to mention the removal of sticky fibrosis. Some surgeons avoid difficult synechiolysis manipulations and opt for transscleral fixation of a foldable PCIOL, implantation of an iris-claw IOL, or an anterior chamber IOL, irrespective of the residual capsulorhexis [5–7]. The disadvantages of each procedure should be considered before abandoning the idea of using a sulcus-based IOL. Transscleral fixation of an IOL requires intricate techniques and is occasionally associated with hypotony, IOL dislocation, and vitreoretinal complications [6]. Iris-claw IOLs can lead to chronic pigment release from the iris, inflammation, and pupil deformation [8]. Anterior chamber IOLs risk corneal endothelial cell decompensation in the long run [9].

Given these surgical dilemmas, we performed a combined procedure of a thorough synechiolysis using vitreoretinal instruments and a sulcus placement of a large-optic and rigid polymethyl methacrylate (PMMA) IOL, CZ70BD (Alcon, Fort Worth, TX, USA), in aphakic eyes with an oversized capsular rim in recent years. The CZ70BD IOL was originally designed for sulcus placement and has eyelets on haptics to facilitate suture fixation [10, 11]. We chose this lens for several reasons: (1) its favorable 7.0 mm optic size for an oversized capsular rim at a diameter close to 7 mm and its 12.5 mm tip-to-tip size; (2) tensile material for stability in the sulcus; and (3) long C-shaped haptics posteriorly angulated 5° to the optic to vault the optic from the iris. Herein, we present the visual and anatomic outcomes in a retrospective case series of aphakic eyes.

## 2. Methods

### 2.1. Patients and Methods

This study was conducted in accordance with the Declaration of Helsinki. All procedures were approved by the ethics committee of Xinhua Hospital, which is affiliated with Shanghai Jiao Tong University School of Medicine, Shanghai, China. Informed consent was obtained from all participants.

A retrospective chart review was performed on consecutive patients who had surgical synechiolysis and then sutureless sulcus placement of a CZ70BD IOL on the preserved large capsulorhexis at the Department of Ophthalmology, Xinhua Hospital, between 2013 and 2015. Clinical records were collected as follows: basic demographics, preoperative best corrected visual acuity, axial length and other available biometric parameters, horizontal white-to-white value (Lenstar LS900®, Haag Streit, Koeniz, Switzerland), preoperative ocular comorbidities, records of intraoperative procedures and videos, intraoperative or postoperative complications, postoperative best-corrected visual acuity, refractive status, and postoperative ophthalmic findings. IOL power calculations were performed using the SRK/T formula adjusted for sulcus fixation as follows: the sulcus IOL power was reduced by 0.5 diopter (D) from the chosen in-the-bag power in the IOL range 9.0 to 17.0 D, by 1.0 D from 17.5 to 28.0 D, and by 1.5 D above 28.0 D [12]. The target postoperative refraction was set as ± 0.5 D for adult emmetropia, at least –3.0 D overcorrection for highly myopic, and based on age for pediatric patients as described by Enyedi et al. [13].

### 2.2. Surgical Techniques

After topical or general anesthesia was administered, the surgical procedures began with two to three limbal side-port paracenteses to introduce in a 20 or 23 G anterior chamber maintainer and other instruments (Supplementary Materials, Video 1).

**Figure 1:**
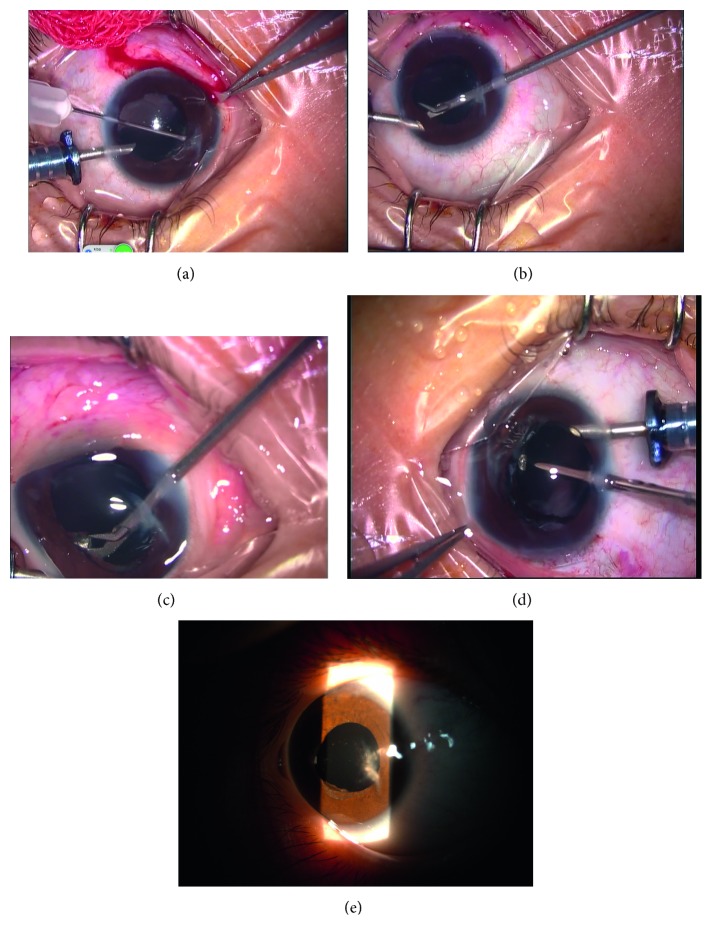


**Figure 2 fig2:**
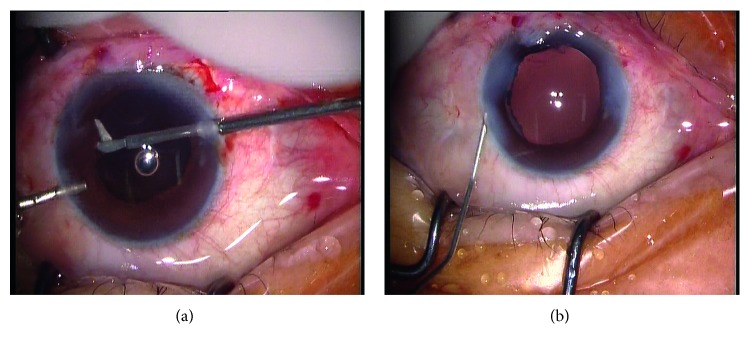


**Figure 3 fig3:**
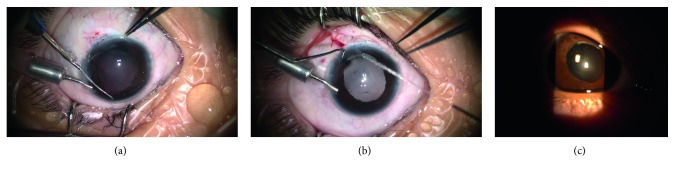


**Figure 4 fig4:**
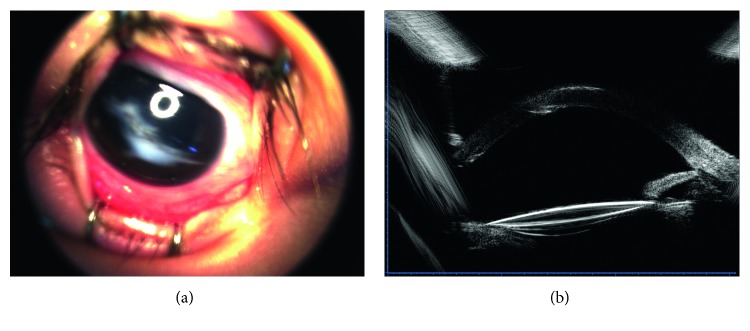


**Figure 5:**
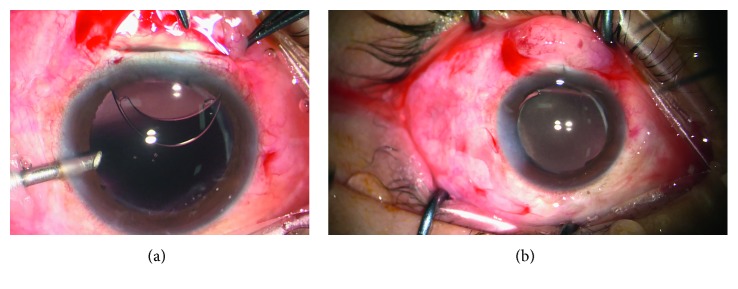


Figures 1 [Fig fig2] [Fig fig3] [Fig fig4]–5 show intraoperative and anterior segment photographs and ultrasound biomicroscopy of patients 2 (Figure 1), 3 (Figure 2), 5 (Figure 3), 6 (Figure 4), and 7 (Figure 5). All patients had surgical synechiolysis to expose the capsulorhexis and then sulcus placement of an Alcon CZ70BD IOL on the oversized residual capsular rim. The arrow points to the capsular edge.

Figures 1(a)–1(d), 2(a), 3(a), and 5(a) show intraoperative photographs taken before the IOL was placed. The arrows point to the preserved capsular rim. Extensive surgical synechiolysis performed in patient 1 is shown in Figures 1(a)–1(d). The 360° iridolenticular adhesion was dissected using a 27 G needle tip and 23 G vitreous scissors, and all fibrosis was removed by a vitreous cutter. After sufficient exposure of the capsulorhexis almost to the ciliary process, the foreign body embedded in the ciliary process appeared, and then it was grasped out smoothly. Synechiolysis procedures in Figures 2(a) and 3(a) also show the effect of dissection and preservation of residual capsule tissue.

Figure 4(a) shows the anterior segment photograph taken by a RetCam fundus imaging system (Clarity Medical Systems, Pleasanton, CA, USA).

Figures 2(b), 3(b), and 5(b) show intraoperative photographs taken after the IOL was placed in the sulcus. The IOL was well centered at the end of the surgery. The IOL optic was partly supported by the residual capsule.

Figures 1(e) and 3(c) show slit-lamp photographs showing that the IOL was well centered at the last visit.

Figure 4(b) shows the ultrasound biomicroscope photograph of patient 6 showing a well-positioned IOL 14 months postoperatively.

Surgical synechiolysis for focal adhesion started with dissection using a 27 G needle. Extensive and sticky iridolenticular adhesion from the pupillary margin almost to the ciliary process was separated using vitreoretinal scissors which moved tangentially to achieve 180°synechiolysis via one corneal paracentesis and then dissected toward the opposite direction via another corneal paracentesis for the other half sector of the synechia. Fibrosis of the iris and capsule was then removed with a vitrector.

Sutureless implantation of a CZ70BD IOL was attempted when sulcus placement of a foldable 3-piece PC IOL was not possible after evaluation of the oversized capsular rim. Following a superior fornix-based conjunctival flap and creation of a 7.5 × 2.0 mm rectangle-shaped sclerocorneal tunnel, starting 1 mm posterior to limbus, a CZ70BD IOL was then inserted into the anterior chamber through the wound with lens-insertion forceps. Passing through the tunnel, the optic was tilted slightly to facilitate sliding of the leading haptic onto the anterior capsular shelf. If necessary, this step was aided by a secondary instrument in the left hand, or iris retractors, which help to expose the underlying capsular edge. Following that, the trailing haptic was oriented into the sulcus as the optic was gently rotated clockwise. If the residual capsular rim was extremely narrow, the IOL was initially inserted onto the anterior surface of the iris, followed by the introduction of the haptic onto the underlying capsular rim one after another. After gentle adjustment, the IOL was oriented in a centered and stable position, with the haptics riding on the wider part of the capsular rim. A suture for fixation was not performed as stabilization and centralization of the IOL was achieved intraoperatively. The scleral wound was then closed with 10-0 nylon-interrupted sutures. The anterior chamber maintainer was removed, and all the wounds were confirmed as tightly sealed. Finally, the conjunctiva and Tenon's fascia were closed over the wound and suture sites.

## 3. Results

Eight eyes from eight consecutive patients who underwent the surgical procedures were included in this study. The clinical characteristics of the patients are listed in Table 1. Seven patients had previously experienced anterior or total vitrectomy, and six patients had their cataract initially removed by lensectomy. Ocular comorbidities of the aphakic eyes were a corneal scar in four eyes, vitreoretinopathies in seven eyes, and posterior luxation of an originally sulcus-based three-piece foldable IOL (Canon-STAAR Preloaded IOL KS-3 (Ai) with a 6.0 mm optic and 12.5 mm tip-to-tip size) in one eye. The mean preoperative axial length was 22.92 ± 1.78 mm. Available horizontal white-to-white values ranged from 11.57 (patient 5) to 12.59 mm (patient 1).

Synechiolysis was performed in all 8 eyes of which 6 eyes received extensive dissections to re-expose the capsulorhexis and ciliary sulcus. The residual capsular rim was noncontinuous and irregular. The size was approximately 7 mm in seven eyes (patients 2 to 8, Figure 1 [Fig fig2] [Fig fig3] [Fig fig4]–5) and even larger in the other eye (patient 1), precluding sulcus placement of an ordinary foldable PC IOL with a 5.5 to 6.5 mm optic. The rigid IOL was placed in the sulcus onto the anterior capsular shelf. The IOL optic, especially the inferior part, was partly secured by a residual capsular rim in seven eyes. In the other eye, the residual capsular rim with a size beyond the IOL optic only supported the IOL haptics. Concurrent surgeries included pars plana vitrectomy in two eyes, limited limbal vitrectomy in one eye, foreign body removal in one eye, and explanation of the original dislocated IOL in one eye (Canon-STAAR Preloaded IOL KS-3 (Ai) placed in the sulcus).

The mean postoperative follow-up period was 33 ± 12 months. Reasonable visual improvement was achieved in all eyes and a best-corrected visual acuity above 0.25 was observed in four eyes. Mild IOL decentration toward the superior direction was detected one month after operation in patient 1 who had a horizontal white-to-white value of 12.59 mm and a capsular rim beyond the size of the IOL optic. Since the IOL position remained stable throughout the follow-up period, repositioning of the IOL was not attempted. For the other seven patients, care was taken to ensure that the IOL optic was partly supported by the residual capsular rim. Consequently, IOLs were well centered throughout the follow-up period (Figure 1 [Fig fig2] [Fig fig3] [Fig fig4]–5, Table 1). In all patients, minor corneal edema with mild anterior chamber inflammation was resolved after the second week with the use of topical corticosteroids. A mild-to-moderate astigmatism was observed in five patients, and an irregular astigmatism at higher diopters was present in the other three patients with a preexisting corneal scar. The mean prediction refractive error calculation was negligible at six months after surgery. No eye experienced chronic corneal edema, elevated intraocular pressure, uveitis-glaucoma-hyphema syndrome, vitreous hemorrhage, or retinal detachment during the follow-up.

## 4. Discussion

We used a PMMA rigid CZ70BD IOL for sutureless ciliary sulcus placement in aphakic eyes with a large capsular rim preserved after synechiolysis. Taking advantage of the residual capsulorhexis, the ideal strategy for aphakic correction in these eyes was the sulcus placement of a PC IOL, which requires less intraocular manipulations, provides for optimal rehabilitation, and reduces intraocular injury.

The first issue for planning a sulcus-based IOL in these aphakic eyes was to reconstruct the ciliary sulcus and maximize the residual capsule support. A preoperative evaluation of sulcus anatomy and diameter by an ultrasound biomicroscope may be underpowered to predict the size of the residual capsular rim before a surgical synechiolysis. Furthermore, any retained adhesion between the iris and the lens capsule may compromise a centered sulcus placement of a PC IOL and result in lateral or anteroposterior instability. Thus, we incorporated various techniques to separate the adhesion between the capsule and iris almost to the ciliary sulcus. An indication for a sulcus-based PC IOL became apparent after the capsular rim was maximally preserved and exposed.

The choice of a sulcus-based PC IOL was considered. Common IOLs for the sulcus are summarized in Table 2. They differ in aspects of size and haptic angulation. In routine situations, we select a sulcus-fixated IOL as follows: (1). anterior capsulorhexis optic capture of a sulcus-fixated foldable intraocular lens on a continuous 5 to 5.5 mm diameter capsulorhexis; (2). a three-piece IOL on a capsulorhexis with a 6 to 6.5 mm diameter. However, in our cases, the oversized capsular rim with a diameter near 7 mm or even larger precluded sulcus placement of a commonly used foldable 3-piece IOL like Alcon MA 50 [3] or Canon-Staar AQ2010 V [1], owing to their 6.5 mm optic size and softness. Still, lateral instability has always been a concern for a soft foldable IOL in the sulcus if the optic cannot be captured under the oversized anterior capsulorhexis. Considering that the mean ciliary sulcus diameter was 12.51 ± 0.43 mm vertically and 12.19 ± 0.47 mm horizontally for cadaver emmetropes, a rigid PMMA IOL with a 12.5 to 13.0 mm overall size may be a better back-up alternative for the ciliary sulcus [1, 3, 14]. Although a CZ70BD IOL only has a 12.5 mm tip-to-tip size, we chose this IOL due to its large optic, rigidity, and posteriorly angulated haptics. In addition, the IOL has built-in eyelets on the haptics for extra suture fixation, which facilitates anchoring one or both haptics to the scleral wall in case of intraoperative IOL decentration. Other larger PMMA IOLs, such as the Alcon CR70BU with a larger overall size of 13.5 mm but without a posteriorly angulated optic, or the Alcon MC60BD with a relatively small optic size of 6.0 mm, were considered less suitable thus not chosen for sulcus placement in our cases.

Favorable visual and anatomic outcomes in our cases provide evidences that our surgical design was reasonable and unwanted intraoperative trauma was minimal. The implantation of IOL was done smoothly on the residual capsulorhexis into the ciliary sulcus when a thorough surgical synechiolysis had been achieved in a minimally invasive but effective way. To our knowledge, there are no clear guidelines regarding the minimum amount of the residual capsule necessary to support a PC IOL in the sulcus. Our experience indicated that IOL haptics should be oriented away from the narrow rim, and the optic, especially the inferior part, should be partly held by a capsular rim with a similar size to the optic. Caution should be taken for eyes with a larger anterior segment as well as a larger capsular rim. In this study, the positional stability of the IOL may be mainly attributed to the similar size of the residual capsular rim compared to the optic size. Superior decentration of the IOL in patient 1 was probably due to the lack of the capsular rim holding the optic and a larger sulcus beyond the tip-to-tip size of the IOL. In this case, suture fixation of one haptic of the IOL would be beneficial. Still, preoperative evaluations of the capsule support and white-to-white values are critical in patients with myopia for proper choice of a sulcus-based IOL, as was the case for patient 7 in our study. Although impossible in our cases, capture of the IOL optic under the anterior capsulorhexis may be a better resolution for the possibility of rotation or lateral subluxation with IOLs placed in the sulcus [15]. Additionally, it was reported that a PC IOL can be integrated into the tissue of the ciliary sulcus, which otherwise may fixate the IOL in the long term [16].

A limitation of the method we used was the occurrence of more pronounced postoperative astigmatism in some patients, owing to a larger scleral tunnel for accommodation of the rigid IOL. In our experience, the surgically induced astigmatism for a 6–7 mm scleral tunnel ranged from 1.0 to 3.0 D. In our cases, a meticulous construction of the incision was made, and the refractive outcomes were acceptable. Three eyes with postoperative astigmatism over 2.0 D may have been partly due to preexisting corneal scars. In conclusion, sulcus placement of a large-optic and rigid CZ70BD IOL may be a reliable and feasible surgical alternative for aphakic correction in eyes with an oversized capsular rim, which is inadequate for a foldable IOL. A preserved capsular rim after surgical synechiolysis, which can partly secure the optic, may be an apparent indication for sutureless sulcus placement of the IOL. The long-term effect and stability of our method should be further evaluated. The comparison to other types of rigid IOLs placed in the sulcus requires further investigation.

## Figures and Tables

**Table 1:** Patients characteristics and surgical outcomes.

Pt	Age (years)/gender	Eye	Preexisting condition	Surgical history (M/Y)	Secondary IOL implantation and concurrent surgeries (M/Y)	FU (M)	BCVA before/after OP(Chinese tumbling E chart)	Al (mm)/horizontal WTW	Prediction error	Astigmatism post (absolute value, diopters)	IOL position	Complication
1	7/M	R	Oversized vitrectorrhexis, corneal scar, ERM	Corneal laceration repair after penetrating injury (9/2012); lensectomy + PPV for cataract and VH (9/2012)	Extensive synechiolysis, sulcus placement of CZ70BD IOL (12/2012)	53	0.08/0.15	22.65/12.59	0.25	2.25	Mild decentration	None
2	7/M	R	Oversized vitrectorrhexis, corneal scar, ocular foreign body, preretinal hemorrhage	Corneal laceration repair after penetrating injury (1/2014); lensectomy + PPV for cataract and VH (1/2014)	Foreign body removal, PPV, extensive synechiolysis, sulcus placement of CZ70BD IOL (7/2014)	16	0.01/0.1	22.06/NA	−1.5	3	Centration	None
3	56/F	R	Oversized CCC	Phacoemulsification + PPV for cataract and VH caused by ocular contusion (4/2013)	Extensive synechiolysis, sulcus placement of CZ70BD IOL (10/2013)	44	0.3/0.5	23.01/12.39	−0.5	1.5	Centration	None
4	3/M	R	Oversized vitrectorrhexis, IOL dislocation	Lensectomy + anterior vitrectomy for congenital cataract (8/2011); sulcus implantation of a foldable IOL (9/2014)	Explanation of original IOL, synechiolysis, and sulcus placement of CZ70BD IOL (9/2014)	32	0.1/0.3	22.7/NA	−0.25	1.5	Centration	None
5	7/M	R	Oversized vitrectorrhexis, corneal opacity, PHPV, optic dysplasia	Lensectomy + anterior vitrectomy for combined PFV (7/2008)	Extensive synechiolysis, extraction of lens remnants, PPV, sulcus placement of CZ70BD IOL (6/2015)	28	0.1/0.25	21.6/11.57	−0.4	1.75	Centration	None
6	2.5/M	R	Oversized vitrectorrhexis, corneal scar	Corneal laceration repair + lensectomy + formation of anterior chamber after ocular blast injury (2/2013)	Extensive synechiolysis, limited vitrectomy, sulcus placement of CZ70BD IOL (6/2015)	26	CF/0.1	22.83/NA	−0.75	2.25	Centration	None
7	39/M	R	Oversized CCC, high myopia	Phacoemulsification + PPV + scleral buckling for RRD (12/2014)	Synechiolysis and sulcus placement of CZ70BD IOL (8/2015)	40	0.8/1.0	27.1/12.19 for horizontal and 12.42 for vertical	0.5	1	Centration	None
8	2/M	R	Oversized vitrectorrhexis, corneal scar	Corneal laceration repair after penetrating injury (7/2014); lensectomy + anterior vitrectomy for cataract (8/2014)	Extensive synechiolysis, sulcus placement of CZ70BD IOL (8/2015)	26	LP/CF	21.4/NA	0.75	1.75	Centration	None
Mean ± SD						33 ± 12		23 ± 2	−0.2 ± 0.7	1.9 ± 0.6		

AL, axial length; BCVA, best-corrected visual acuity; CCC, continuous curvilinear capsulorhexis; CF, counting fingers; ERM, epiretinal membrane; IOL, intraocular lens; LP, light perception; PFV, persistent fetal vasculature; PPV, pars plana vitrectomy; RRD, rhegmatogenous retinal detachment; VH, vitreous hemorrhage; M, male; F, female; R, right; IOL, intraocular lens; FU, follow-up visit period; M, months; Y, years; WTW, white-to-white.

**Table 2:** Representative types of IOLs used for sulcus placement in literatures [1, 3].

Type		Size, optic/overall (mm)	Haptic angulation
*Foldable*			
Three-piece silicone optic	Staar AQ 2010V	6.3/13.5	Yes
Three-piece acrylic	Alcon MA50	6.5/13.0	Yes
Three-piece acrylic	Alcon MA60AC	6.0/13.0	Yes

*Rigid*			
Single-piece PMMA	Alcon CZ70BD	7.0/12.5	Yes
Single-piece PMMA	Alcon MC60BD	6.0/13.5	Yes
Single-piece PMMA	Alcon CR70BU	7.0/13.5	No

IOL, intraocular lens; PMMA, polymethyl methacrylate.

## Supplementary Materials

Supplementary Materials Video 1: the surgical procedures of patient 5 who had synechiolysis of iridocapsular adhesion and sulcus placement of a rigid intraocular lens on an oversized residual capsular rim. The 7-year-old boy had aphakia in the right eye due to primary lensectomy and anterior vitrectomy for combined persistent fetal vasculature in July 2008.
